# Patient safety: a new basic science for professional education

**DOI:** 10.3205/zma001229

**Published:** 2019-03-15

**Authors:** Albert W. Wu, Isolde M. Busch

**Affiliations:** 1 Johns Hopkins Bloomberg School of Public Health, Department of Health Policy and Management, Baltimore, MD, United States; 2 University of Verona, Department of Neuroscience, Biomedicine and Movement Sciences, Section of Clinical Psychology, Verona, Italy

## Introduction

“Patient safety is a core attitude and thus needs to be introduced early and then reinforced throughout postgraduate education and continuing professional development.” (Stefan Lindgren, President of the World Federation for Medical Education)

Beginning in the 1990s, studies of hospital safety and quality from around the world have consistently found problems with patient safety and quality [1]. There has been a notable increase in awareness of the problem, with major efforts in the past two decades to improve the safety of medical care. A study conducted for the World Health Organization found that seven types of adverse events cause 43 million injuries a year, making preventable harm the world’s twentieth most common cause of overall morbidity and mortality [2]. Others have suggested that medical errors are even more common [3]. A chilling statistic from WHO was that in high income countries, on average, one of every ten patients hospitalized suffers a serious, preventable adverse event [4].

Although patients continue to be harmed by health care, there has been some progress [5]. Since 2000, it has become widely understood and accepted that “it’s the system” – it is the health care system that creates hazards and harm, and that also creates patient safety, rather than individual providers [6]. However, there is a deeply seated, pernicious habit for people, the public and health care managers to blame specific medical errors exclusively on individual health professionals. On the other hand, it is certainly true that individuals are an integral and indispensable component of the health care system. Individuals also act as members of teams, and interact with other parts of the system [7]. Individual must feel they are accountable too. If the balance of accountability swings too far, and we rely entirely on searching for systems solutions, the important process of changing individual behaviors will be lost [8]. 

Regardless of whether you take an individual or system perspective on the causation of medical error, there is a need to educate clinicians on how to deliver safer care. We believe that patient safety should be a new basic science for professional education. To accomplish this, major reforms are needed in health professions education. However, we appreciate that there are challenges associated with incorporating patient safety into education and training. 

This special issue on patient safety in medical education in Germany represents an important step to increasing awareness of patient safety as an important element in the training of health professionals. The papers in this issue help to advance the field, in both education and in research on education.

## The problem

Schools in the health professions, including medicine, nursing, pharmacy, dentistry and others, provide limited education on patient safety. In medicine, the traditional curricular focus is on basic science and medical knowledge. Residency and other post-graduate training add a focus on technical expertise. Other health professions, including nursing, pharmacy and health technology maintain the primary focus on acquiring facts and knowledge. None pay sufficient attention to the key concepts, attitudes and skills necessary to practicing safely and spurring improvements in care. 

In addition to the lack of basic knowledge and skills, the prevailing culture and work environment in hospitals and other health care organizations work against many of the prerequisites for safe practice. In many organizations there is a pernicious culture of shame, blame and punishment surrounding medical errors, and a deny-and-defend stance in response to patients and families. A “hidden curriculum” reflecting this culture sabotages attempts at classroom education [9]. Together, these conditions prevent awareness, taking action, and learning from errors.

To address these gaps, health professions schools and training programs will need to refocus their goals, away from the mere acquisition of knowledge and facts. Programs will need to make room in the curriculum for new concepts, attitudes, behaviors and skills, and provide opportunities for trainees to implement them in practice.

## Examples of education

There is accumulating evidence that education can help to improve patient safety and health care quality. Safety curricula are generally popular among trainees and have resulted in increased knowledge of safety and quality improvement (QI) concepts, and led to improvement in care processes [10].

Medical schools are beginning to introduce patient safety training into the undergraduate curriculum [11], [12]. At the Johns Hopkins University in the U.S., a 10-hour curriculum was instituted for first year medical students, and showed improvements in knowledge and attitudes, including future commitment to patient safety [13]. This has been followed by a 3-day curriculum for second year medical students, shortly before they transition from classroom to clinical wards, including lectures and hands-on experiences, which showed advances in knowledge, self-efficacy and systems thinking [14]. It is noteworthy that this curriculum consistently receives the highest evaluations from students among all of the special topics taught in the second year. 

In the UK, a 5-hour curriculum for senior medical students on understanding error in health care was shown to have improved knowledge [11]. 

Aiming to implement patient safety curricula in the medical education in German-Speaking countries, the committee for Patient Safety and Error Management of the German Association for Medical Education introduced in 2016 a Learning Objective Catalogue addressing patient safety topics and error management in Medical Education. This catalogue serves as basis for a deepened discussion of patient safety issues among medical faculties and as disciplinary and content-related orientation guide for embedding patient safety teaching courses into existing medical curricula [15], [16].

In practice, studies have found education to benefit patient outcomes. Aiken and colleagues showed that hospitals in the U.S. with higher levels of nursing education had lower patient mortality rates [17], [18]. Berry and colleagues demonstrated that improved safety culture and teamwork climate were associated with decreases in patient harm and hospital mortality [19].

## What should be taught?

The Institute of Medicine’s groundbreaking report *To Err Is Human* in 1999 [6] and subsequent publications have influenced recommendations worldwide to promote safer health care. These recommendations have favored competencies over content, with the goal of changing the behavior of health professionals.

Competencies encompass patient safety within the broader domain of practice. In its report Patient Safety Achieving a New Standard for Care, the Institute of Medicine identified 5 core competencies which all health professional should be able to demonstrate [20]. These included the provision of patient-centered care, the ability to work in interdisciplinary teams, employment of evidence-based practices, application of quality improvement concepts, and utilization of informatics.

Several influential groups and authoritative bodies have launched efforts to identify sets of competencies important to promote safer health care practice [20], [21], [22], [23], [24], [25]. 

The American College of Graduate Medical Education and the American Board of Medical Specialties [22] identified competencies within the domains of patient care, medical knowledge, practice-based learning and improvement, interpersonal and communication skills, professionalism, and systems-based practice. These are shown in table 1 (Tab. 1).

The World Health Organization Patient Safety Programme identified 11 key topics to be covered. This list was initially based on the Australian Patient Safety Education Framework [26], [27]. 

The first topic regards the concept and definition of **patient safety** itself. **Human factors** describe the interaction of workers within the work system of health care, and how specific internal factors (knowledge, skills) and external factors (stress, ineffective communication, production pressure) may be associated with medical errors and adverse events. **System** failures and patient harm can result from factors originating from multiple levels within the health care system. These system levels include the patient, the task, the individual, the team, tools, management and organization. Communication and **teamwork** involve patients and their carers, as well as interdisciplinary collaboration to ensure high quality care. The ability to see systems and failures within them, and to communicate incidents to colleagues are crucial to **learning from errors.** The ability to use **quality improvement** tools allows closing the loop after these events. **Engaging with patients** and their caregivers is essential to optimizing safety. This includes behaving ethically and appropriately in **managing clinical risk** and being open with patients about medical errors. **Infection control** identifies potential hazards and prevents health care associated infections particularly through the application of universal precautions. Invasive procedures are a particularly high-risk part of health care; harm can be reduced through the judicious use of checklists and standard operating procedures. **Medication safety** addresses the ubiquitous risks associated with all of the phases of medication use, particularly for different age groups, high hazard medications and transitions of care. 

There is an increasing number of patient safety curricula available, and reviews of their successes and challenges [10], [28], [29], [30], [31], [32], [33], [34]. The World Health Organization developed a curriculum guide to provide medical students with essential patient safety lessons to allow them to practice safely [26]. It includes a teacher’s guide, and a comprehensive, ready-to-use, topic-based programme with a full set of slides. 

Recognizing that other professionals provide the majority of care to people in all countries, the medical student curriculum was followed closely by a more general multi-professional patient safety curriculum guide. This aimed to aid in the implementation of training in inpatient safety, including in the fields of midwifery, nursing and pharmacy, dentistry, and medical technology [35]. 

## Challenges to implementation

It is sometimes said that the hardest thing to do in academic medicine is to get a new course added to the medical school curriculum. Universities are well known for their professional bureaucracy, and this includes resistance to change. There is a tendency to preserve existing organizational structure, even when it is obvious that it fails to serve institutional goals. 

Barriers have been noted to making changes to higher education in general, related to attitudes, existing structures, and resources [36], [37]. Published literature suggests that it can also be challenging to introduce patient safety into health professions schools [38].

Human factors barriers to making changes to higher education in general include:

1. Lack of awareness (including lack of interest, engagement, involvement), support, professionalism, policy making and recognition
2. Unsupported structure, conservative disciplinary organization of higher education, inefficient communication, resistance to change, overcrowded curriculum, focus on content-based learning
3. Need for more resources including funding, work pressure and lack of time, lack of access to information, lack of consistent legislation and lack of physical place [36]

There are specific challenges to incorporating patient safety into health professions education. These include

1. lack of awareness,
2. lack of agreement, including the hidden curriculum,
3. lack of engagement and involvement,
4. lack of leadership,
5. the discipline-based structure of medical science and health care,
6. resistance to change,
7. overcrowded curriculum,
8. historical focus on content-based learning,
9. lack of know-how and support for educators, including funding
10. competing work pressures and lack of time,
11. evidence gaps in best practices.

Lack of awareness and lack of agreement are recognized barriers to physician adherence with guidelines and behavior change [39]. Course directors are reluctant to accept the need for patient safety science [40]. Many need to be convinced of the importance of this subject in relation to other subjects for students e.g., foundational sciences like anatomy, physiology, and biochemistry. 

A hidden curriculum reflecting “real world practice” is ingrained in the culture and behavior of health care organizations. This hidden curriculum perpetuates hierarchies of authority and unprofessional behavior, sabotages teamwork, and reinforces paternalistic attitudes towards patients [9]. Students and trainees witness how their more experienced colleagues behave, contrary to the lessons they might have been taught in the classroom. These factors contribute to a lack of engagement and involvement by faculty members, which can be exacerbated by the lack of visionary and enabling leadership to advance the patient safety agenda.

The discipline-based structure of medical schools is itself a barrier [40]. Faculty members in individual departments may be reluctant to relinquish space on the curriculum and the status it implies. In a recent personal experience at our own institution, an attempt to teach medical and nursing students together about patient safety was frustrated by conflicting calendars for the respective schools.

Professional schools are already struggling with curricula that are densely packed. There is a limited amount of clinical time, which may reduce the opportunities for students to be exposed to common patient safety issues. Opportunities for interdisciplinary training in practice settings are even more limited [41]. In addition, faculty are accustomed to providing content-based rather than competency-based learning. There may be an insufficient number of faculty to teach in this area, and existing faculty may be uncomfortable teaching material outside of their own discipline and expertise [42].

Thus, top leaders in professional schools and academic medical centers have an important role to play in making successful curricular change. This requires creating a milieu in which change can be accepted and made. Insufficient support in terms of time, financing, and guidance are important barriers to adoption of reforms and their implementation [43].

## What should be done

To make changes in curriculum, instill competencies and culture in trainees, and ultimately affect changes in behavior, action will be needed on the part of multiple stakeholders in health professions education and health care organizations.

For an organization embarking on this kind of curricular change, communication and engagement of educators and staff are essential from the start [44], [45]. It is important to let people know the rationale for the proposed changes, and the process of change that will occur. It is crucial to provide professional development opportunities to staff who will be involved. This should include relevant training and activities that will allow them to engage in the change process. 

The Lucian Leape Institute of the National Patient Safety Foundation convened an expert group that produced recommendations for improving education in patient safety [46]. Although they were developed in the US for medical school reform, most of these recommendations are widely applicable to the international context and for other professional schools. The recommendations focus first on developing learning cultures that emphasize safety, professionalism, collaboration and transparency. There is an emphasis on promoting interpersonal skills and interdisciplinary teamwork. Resources should be provided to support faculty development of the skills needed to deliver the curricula. Changes should extend to selecting students with the attributes that reflect these new competencies. Patient safety should be conceptualized as a science, and undergraduate professional education should focus on core competencies within the domains identified earlier in this editorial. This learning should extend beyond undergraduate and specialty training, leading to lifelong learning. National accreditation requirements should be aligned with the achievement of these competencies. The impact of this new set of educational priorities should be evaluated, and financial and other incentives should be aligned to support the changes. Many of the recommendations are directed at top university leaders, and at even higher-level external leaders in education ministries and accrediting bodies, as these individuals play important roles in managing change [47].

There is a variety of tools and strategies that can be deployed. Detailed discussion of these is beyond the scope of this paper. However, these include both high tech and lower tech simulation [48], [49] and also the use of standardized patients. Training in the use of standardized tools to improve teamwork and communication, such as TeamSTEPPS and the Comprehensive Unit Based Safety Program (CUSP) can be useful to improve knowledge, attitudes, and outcomes [50], [51], [52].

Traditional role modeling remains crucial as an essential element of teaching. This is particularly important for imparting values and behaviors that support a culture of safety and optimal learning, and to support both the prevention and handling of errors. Partnering whenever possible with patients is also an important part of education [53]. 

There are excellent basic textbooks [54], [55], as well as a growing bank of free on-line resources to provide information, guidance and training in patient safety [56], [57], [58], [59], [60].

## Conclusion

It is a worldwide imperative to prepare the health care work force to deliver safer care. We believe that patient safety should be regarded as a new basic science for health professions education. However, the translation of patient safety science into safe practice is also a highly applied activity. Major reforms will be needed to incorporate patient safety into the curricula of professional schools and training programs. These organizations will need to redirect their focus away from the mere acquisition of knowledge to developing competencies and changing behavior.

The new curriculum will need to include competencies related to providing patient-centered care, working in interdisciplinary teams, using evidence-based practices, and applying quality improvement concepts. These competencies involve changing how students see, and changing attitudes and skills. We would like students to be able to see individual safety problems with system lenses, and be able to identify and test potential solutions.

We are aware that there are challenges associated with integrating patient safety into education and training. A major barrier is the prevailing culture of shame, blame, and denial about medical errors. The hidden curriculum competes with attempts to create a culture of safety and allow optimal learning.

Action will be needed from multiple stakeholders in health professions education and health care organizations. Communication from top leaders and transparency throughout the organization are needed in the entire process. Coordination will also be needed to give students opportunities to practice their new skills in real world settings.

We now have sufficient tools for any organization to make a good start. There is still much to learn, such as effective strategies to educate trainees in multidisciplinary and practice-based settings, and how to adapt materials to fit the local context. Innovations are still needed, and building research and evaluation into early efforts will help us arrive more quickly to the goal of making patients safer.

## Competing interests

The authors declare that they have no competing interests.

## Figures and Tables

**Table 1:**
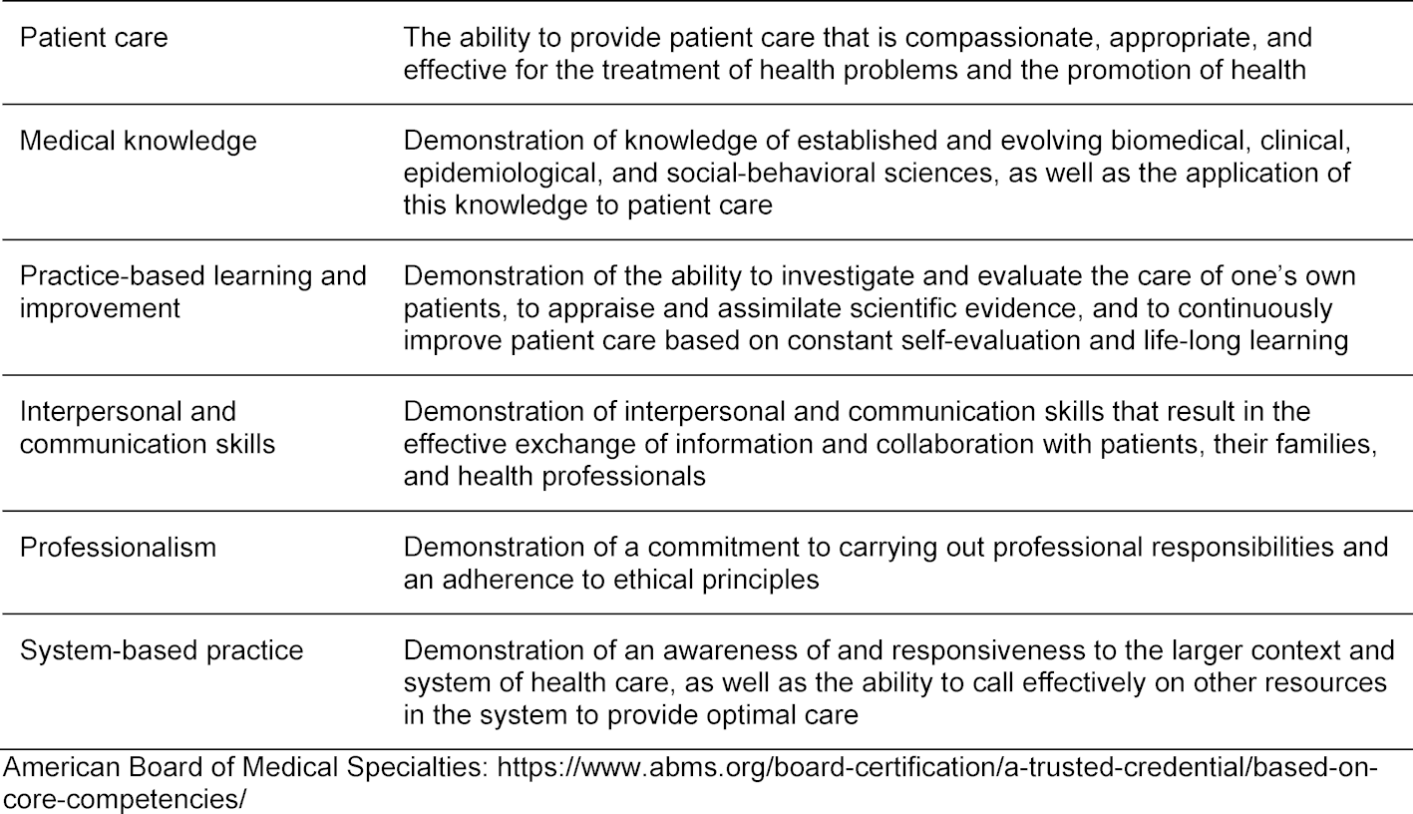
Core Competencies and Criteria for Maintenance of Certification as defined by the American Board of Medical Specialties
